# Minimal Clinically Important Difference and Substantial Clinical Benefit After Carpal Tunnel Release Using the Quick Disabilities of the Arm, Shoulder, and Hand (QuickDASH) and Mayo Wrist Scores: A Prospective Observational Study

**DOI:** 10.7759/cureus.105359

**Published:** 2026-03-17

**Authors:** Ammar Al Balushi, Abdullah Al Lawati, Moath Shummo, Marwa Al Badi, Asmaa Al Furqani, Ahmed M Al Wahaibi, Waleed Al-Nizwani, Nawaf A Al-Muqaimi, Ahmed Al Ghaithi

**Affiliations:** 1 Orthopaedic Surgery, Oman Medical Specialty Board (OMSB), Muscat, OMN; 2 Plastic and Reconstructive Surgery, Sultan Qaboos University Hospital, Muscat, OMN; 3 Orthopaedic Surgery, Khawlah Hospital, Muscat, OMN; 4 Orthopaedic Surgery, Sultan Qaboos University Hospital, Muscat, OMN

**Keywords:** carpal tunnel syndrome, disability evaluation, median nerve decompression, patient-reported outcome measures, surveys and questionnaires, treatment outcome

## Abstract

Background

Carpal tunnel syndrome is a neuropathic compressive condition caused by median nerve compression as it travels into the wrist through the carpal tunnel. Validated patient-reported outcome measures such as the Quick Disabilities of the Arm, Shoulder, and Hand (QuickDASH) and the Mayo Wrist Score are commonly used to assess outcomes following carpal tunnel release; however, limitations remain in interpreting whether observed score changes are clinically meaningful from the patient’s perspective. The primary objective of this study was to determine the minimal clinically important difference (MCID) and substantial clinical benefit (SCB) for the Mayo Wrist Score after isolated carpal tunnel release, and secondarily to contextualize postoperative QuickDASH change using previously reported clinically meaningful thresholds.

Methodology

This prospective study included adult patients undergoing isolated carpal tunnel release at two tertiary hospitals. QuickDASH and Mayo Wrist Scores were recorded preoperatively and at the six-month follow-up. MCID and SCB were calculated using anchor-based and distribution-based methods, with receiver operating characteristic curve analysis primarily applied to the Mayo Wrist Score*.*

Results

A total of 92 patients were enrolled, of whom 73 completed the six-month follow-up. The anchor-based MCID for the Mayo Wrist Score was 11.5 points (area under the curve (AUC) = 0.77), while the SCB threshold was 13.5 points (AUC = 0.91). Significant postoperative improvements were observed in both QuickDASH and Mayo Wrist Scores.

Conclusions

Establishing MCID and SCB values for the Mayo Wrist Score and contextualizing change in the QuickDASH score provides a clinically meaningful framework for interpreting patient-reported outcomes following carpal tunnel release.

## Introduction

Carpal tunnel syndrome (CTS) is caused by median nerve compression as it travels into the wrist through the carpal tunnel [1,2]. It is a common condition affecting about 5-6% of the general adult population, with an incidence of approximately 4 cases per 1,000 individuals per year, making it the most common compressive neuropathy of the upper extremity [3-5]. Nonoperative and operative options for CTS management have been extensively studied.

In the past, research focused primarily on the objective assessment of CTS surgery rather than validated patient-reported outcome measures. In addition, outcome assessment was often subject to observer bias due to the lack of standardized scoring systems. Therefore, to overcome this issue, validated outcome measurement tools were proposed to assess disability associated with CTS, guide treatment decisions, and evaluate the success of interventions [6]. Moreover, these tools support evidence-based practice and improve the quality of healthcare delivery. Commonly used validated instruments include the Disabilities of the Arm, Shoulder, and Hand (DASH), the Boston Carpal Tunnel Questionnaire, the QuickDASH, and the Mayo Wrist Score [7,8].

Despite the availability of these tools, limitations remain in interpreting whether observed changes are clinically meaningful from the patient’s perspective. As a result, the concept of the minimal clinically important difference (MCID) was developed [9]. This concept was initially introduced by Jaeschke et al. in 1989 and defined as the slightest change in an outcome measure that patients perceive as important and worthwhile [10]. Furthermore, MCID serves as a benchmark to determine the effectiveness of a treatment and to assess whether changes in patient-reported outcome scores represent a meaningful clinical improvement [11]. Establishing MCID values also facilitates comparisons across studies and helps determine appropriate sample sizes for future clinical research. Distribution-based and anchor-based methods are the primary approaches for calculating MCID values [12]. PROMIS-based upper-extremity instruments have also been used to assess clinically meaningful change and establish MCID values [13].

Relatively limited work has been conducted to establish MCID values for commonly used hand-specific patient-reported outcome measures following carpal tunnel release, particularly in Arab and Middle Eastern populations. Previously reported MCID thresholds for carpal tunnel and related upper-extremity outcome measures have varied according to the instrument and study population. In patients undergoing carpal tunnel release, Kazmers et al. reported a distribution-based QuickDASH MCID of 10.4 points, while later work in a non-shoulder hand and upper-extremity population reported QuickDASH MCID values of approximately 10.2-10.3 points [3,13]. These findings suggest that clinically meaningful change is instrument-specific and method-dependent, supporting the need to establish interpretable thresholds for other commonly used measures such as the Mayo Wrist Score. Therefore, this study aimed to better define the clinical outcomes of carpal tunnel release as perceived by patients by establishing MCID and substantial clinical benefit (SCB) thresholds using validated outcome instruments. The primary aim of this study was to calculate the MCID and SCB for the Mayo Wrist Score following carpal tunnel release, while interpreting QuickDASH changes using established clinically meaningful thresholds.

## Materials and methods

Study design and sample size

This study was a prospective observational study of patients diagnosed with CTS based on history, clinical examination, and nerve conduction studies. Patients were evaluated in the day-care unit and scheduled for carpal tunnel release at two tertiary medical care hospitals (Sultan Qaboos University Hospital and Khoula Hospital) between August 2022 and August 2024, with a total study duration of two years. Ethical approval was obtained from the Research Committee of Khoula Hospital (approval number: MOH/DGKH/REC/24/28550). All participants provided written and verbal informed consent before enrollment.

The estimated sample size was 50 patients, with an assumed statistical power of 90% and an alpha level of 5%, consistent with prior studies evaluating MCID using anchor-based and receiver operating characteristic (ROC)-based methods. Patients who met and fulfilled the inclusion criteria were enrolled and followed prospectively.

Inclusion and exclusion criteria

The inclusion and exclusion criteria of the study are presented in Table 1.

**Table 1:** Inclusion and exclusion criteria.

Inclusion criteria	Exclusion criteria
• Age ≥18 years	• Language difficulties
• Isolated carpal tunnel release	• Cognitive/Psychiatric impairment preventing consent
• Provided informed consent	• Cervical radiculopathy or other neurological disorders
• Completed pre- and postoperative validated outcome measures	• Previous hand trauma or major musculoskeletal disorder affecting hand function
	• Subsequent upper-extremity surgery during the study period
	• Alternative treatment or revision surgery planned
	• In bilateral cases, only the first operated hand was included

Surgical intervention

All surgical procedures were performed as day-case surgeries under local anesthesia using a tourniquet, without deviation from the standard open surgical technique for carpal tunnel release. A classic surgical incision was made at the intersection of Kaplan’s cardinal line and the radial border of the fourth ray, extending toward the wrist crease. Dissection was carried through the subcutaneous tissue and palmar fascia until the transverse carpal ligament (TCL) was identified.

All critical anatomical structures were protected throughout the procedure. Under direct visualization, the TCL was released, and the median nerve was decompressed. All patients received standardized postoperative care, including suture removal at 10-14 days and routine outpatient follow-up. During follow-up visits, patients underwent clinical assessment and completed the assigned outcome measures.

Data collection and outcome assessment

Validated patient-reported outcome measures were used for clinical evaluation, including the QuickDASH questionnaire and the Mayo Wrist Score. The QuickDASH consists of 11 items that assess difficulty performing physical activities related to upper-extremity function [14,15]. Each item is scored on a five-point Likert scale, with total scores ranging from 0 to 100, where higher scores indicate greater disability. A minimum of 10 completed items was required for score validity.

The Mayo Wrist Score consists of four domains assessing pain, range of motion, grip strength, and functional status, with a total score ranging from 0 (worst) to 100 (best) wrist function. Both outcome measures were administered preoperatively at admission and postoperatively at the six-month follow-up. At follow-up, an anchor-based transition question was administered to assess perceived change following surgery. Patients were asked to rate their overall condition using a five-point Likert scale: “much improved,” “slightly improved,” “no change,” “slightly worse,” or “much worse” [16,17].

MCID and SCB determination

The MCID was determined using both anchor-based and distribution-based methods. For the anchor-based analysis, patients reporting “slightly improved” were classified as having achieved the MCID, while patients reporting “no change” were classified as unimproved. Patients reporting “much improved” were excluded from MCID estimation, as their improvement exceeded the minimal threshold. For distribution-based analysis, the MCID was set at 0.5 times the baseline standard deviation, corresponding to a moderate effect size [18].

ROC curve analysis was used to determine optimal cutoffs for MCID and SCB for the Mayo Wrist Score. The MCID cutoff distinguished between improved and unimproved patients, while the SCB cutoff distinguished between “much improved” and non-substantial improvement groups [19]. The area under the curve (AUC) was used to assess discriminatory ability, with values ≥0.7 considered acceptable and ≥0.8 considered excellent [20]. ROC-based MCID and SCB analyses were applied to the Mayo Wrist Score due to its clinician-graded structure and clear directional interpretation, while QuickDASH was interpreted using previously established MCID ranges from the literature.

Statistical analysis

Baseline demographic variables, including age, sex, comorbidities, hand dominance, nerve conduction study grade, occupation type, and operated side, were recorded. Continuous variables were summarized using means and standard deviations, while categorical variables were presented as frequencies and percentages.

Preoperative and postoperative QuickDASH and Mayo Wrist Scores were compared using paired t-tests. Differences in change scores across anchor-based response groups (“much improved,” “slightly improved,” and “no change”) were analyzed using one-way analysis of variance (ANOVA). Post hoc comparisons were performed when appropriate.

All statistical analyses were performed using SPSS software version 29 (IBM Corp., Armonk, NY, USA). ROC curve analysis was conducted to determine optimal cutoff values for MCID and SCB. The Youden index was used to identify optimal thresholds. The AUC, sensitivity, specificity, and associated p-values were calculated. A two-sided p-value <0.05 was considered statistically significant.

## Results

**Figure 1:**
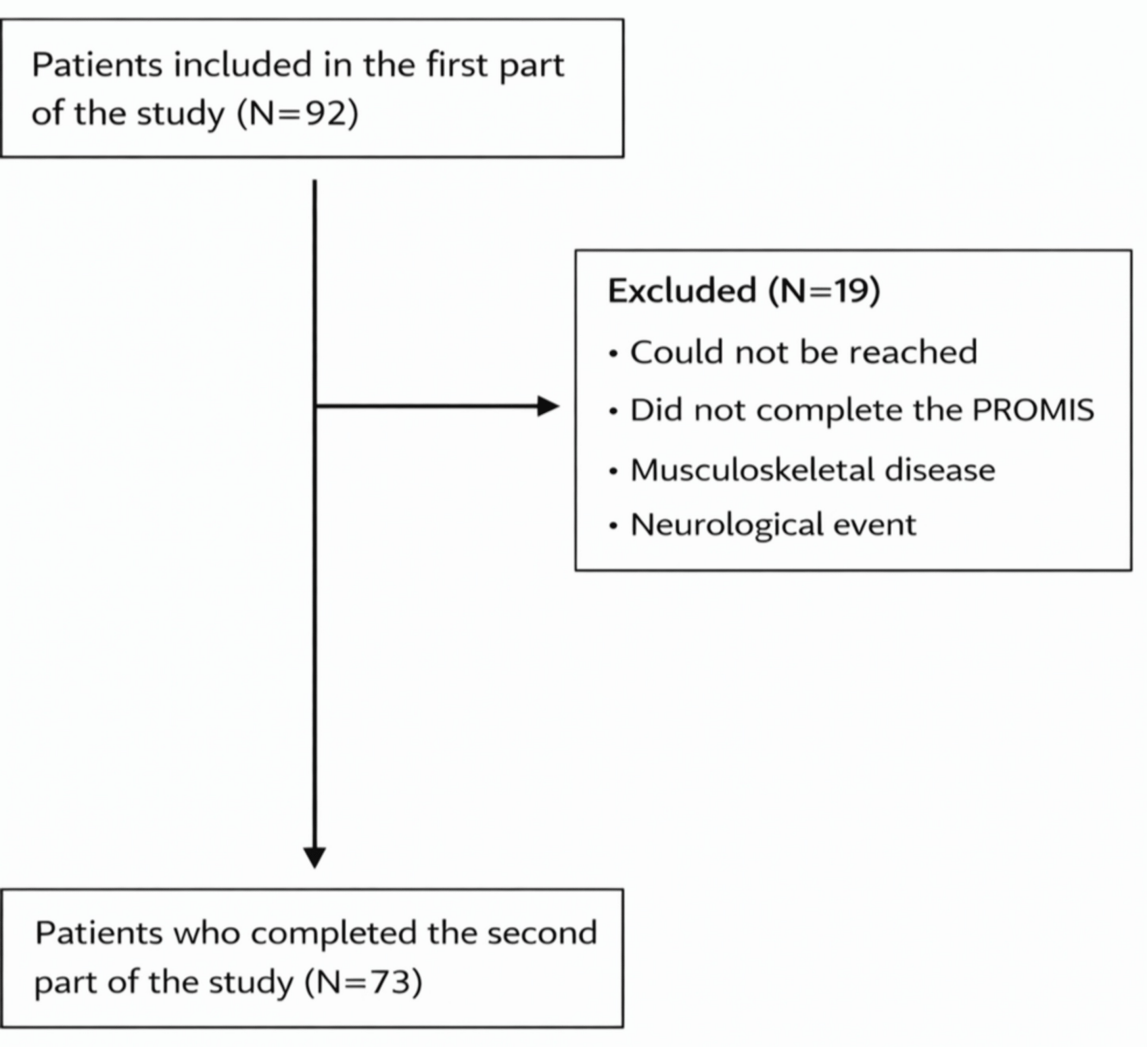
Patient recruitment diagram.

A total of 92 patients were enrolled in the first part of the study. A total of 19 (20.7%) patients were excluded before final analysis for various reasons, including undergoing surgery outside the study centers, relocation, unavailability of the evaluating physicians, failure to complete postoperative patient-reported outcome measures, development of musculoskeletal disease, or intercurrent neurological events. Of the 92 enrolled patients, 73 (79.3%) completed the six-month follow-up and were included in the final analysis (Figure 1).

The mean age of the study population was 47.6 years, and 62 (84.9%) participants were female. Baseline demographic characteristics, including region of residence, treating hospital, comorbidities, hand dominance, and operated side, are summarized in Table 2.

**Table 2:** Demographic characteristics of the study population (n = 73).

Variable	n (%)
Age (years)	
Mean	47.6
Gender	
Male	11 (15.1)
Female	62 (84.9)
Region	
Muscat	45 (61.6)
Other regions	28 (38.4)
Hospital	
Khoula Hospital	68 (93.1)
Sultan Qaboos University Hospital (SQUH)	5 (6.9)
Comorbidity	
Present	29 (39.7)
Absent	44 (60.3)
Hand dominance	
Right	65 (89.0)
Left	8 (11.0)
Operated side	
Right	44 (60.3)
Left	29 (39.7)

Mean preoperative and postoperative outcome scores demonstrated significant improvement following surgery. The Mayo Wrist Score improved from a mean of 33.7 preoperatively to 76.3 at the six-month follow-up. In contrast, the QuickDASH score improved from a mean of 65.8 to 19.0, indicating a substantial reduction in disability (p < 0.01 for both measures) (Table 3).

**Table 3:** Change in QuickDASH and Mayo Wrist Scores stratified by anchor-based patient response. Note: P-values reflect comparisons across anchor response groups. QuickDASH: Quick Disabilities of the Arm, Shoulder, and Hand

Anchor response	n	QuickDASH	Mayo Wrist Score
Much improved	42	54.6	51.3
Slightly improved	26	39.7	34.4
No change	5	17.6	12.0
P-value		<0.01	<0.01

At follow-up, 42 (56.8%) patients reported being “much improved,” 26 (35.1%) “slightly improved,” and five (6.8%) “no change.” No patients reported worsening of symptoms. Overall, 68 (93.2%) patients reported postoperative improvement.

When stratified by anchor response, patients who reported greater perceived improvement showed larger changes in outcome scores. Patients reporting being “much improved” showed a mean improvement of 54.6 points in QuickDASH and 51.3 points in the Mayo Wrist Score, whereas those reporting “slightly improved” demonstrated mean improvements of 39.7 points and 34.4 points, respectively. Patients reporting “no change” showed more minor improvements (p < 0.01 across groups) (Table 3).

**Figure 2:**
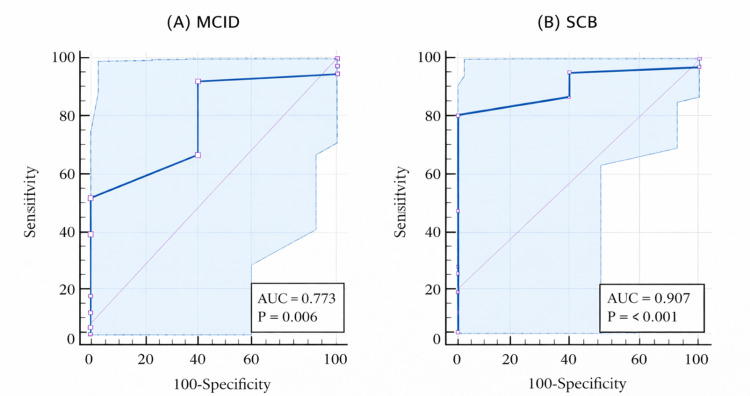
ROC curves for MCID and SCB of the Mayo Wrist Score. MCID: minimal clinically important difference; SCB: substantial clinical benefit; AUC: area under the curve; ROC: receiver operating characteristic

**Figure 3:**
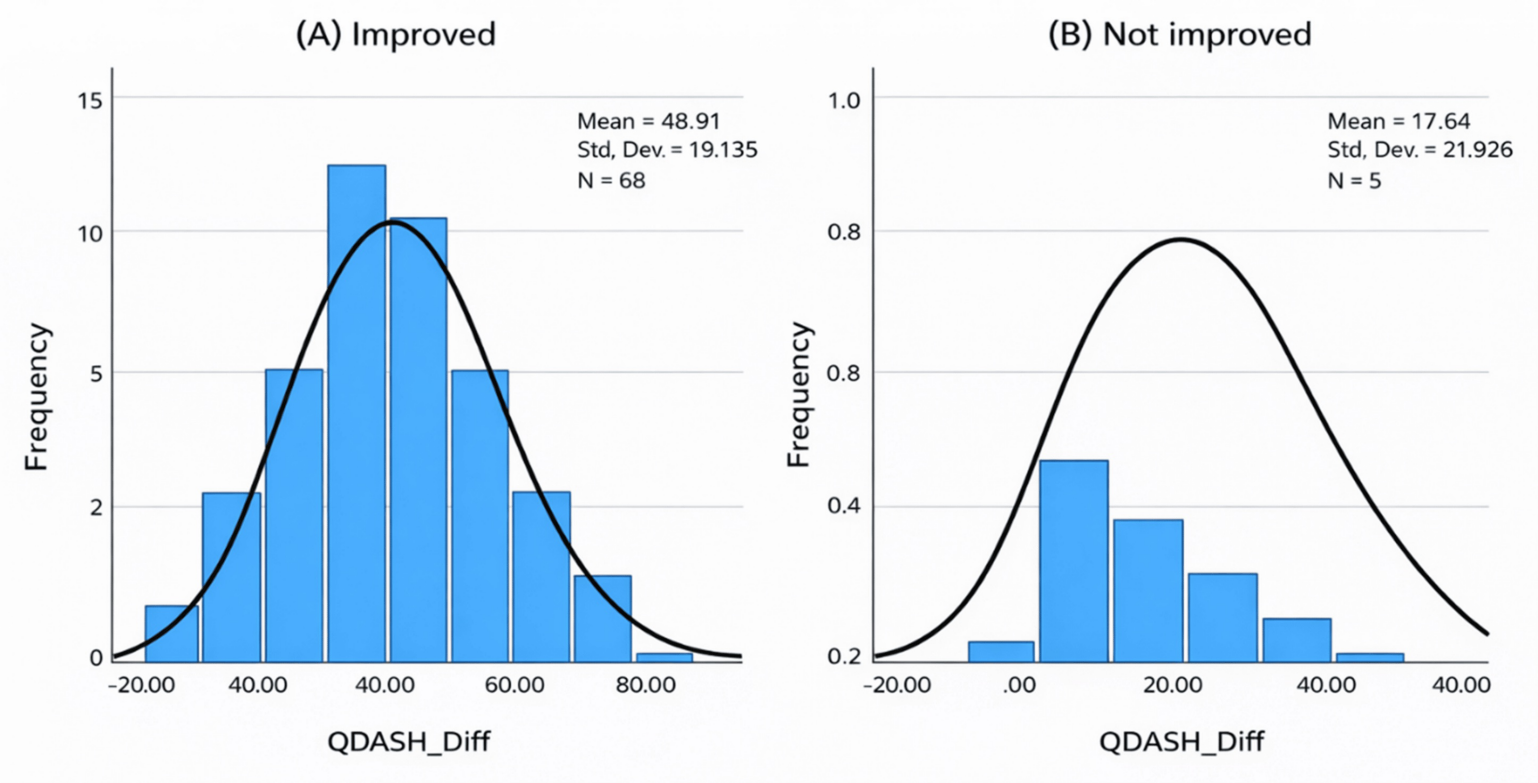
Distribution of change in QuickDASH scores stratified by anchor-based improvement. QuickDASH: Quick Disabilities of the Arm, Shoulder, and Hand

The anchor-based MCID was calculated by comparing patients who reported being “slightly improved” with those reporting “no change” and was 11.5 points, with an acceptable AUC of 0.77 (95% confidence interval (CI) = 0.57-0.97). The SCB, calculated for patients who reported being “much improved,” was 13.5 points, demonstrating excellent discrimination with an AUC of 0.91 (95% CI = 0.84-0.98) (Table 4). ROC-based MCID and SCB analyses were performed for the Mayo Wrist Score. The ROC curves illustrating the optimal cut-point analysis for both MCID and SCB are shown in Figure 2 and Figure 3.

**Table 4:** Anchor-based MCID and SCB for the Mayo Wrist Score. MCID: minimal clinically important difference; SCB: substantial clinical benefit; AUC: area under the curve

	Value	Sensitivity	Specificity	AUC	P-value
MCID	11.5	50	100	0.77	<0.0061
SCB	13.5	79.4	100	0.91	<0.0001

## Discussion

In this study, the MCID for the Mayo Wrist Score was determined to be 11.5 points, while changes in QuickDASH scores were interpreted in relation to previously reported clinically meaningful thresholds. The Mayo Wrist Score demonstrated an acceptable discriminatory ability, with an AUC of 0.77, for differentiating between improved and unimproved patients, with the selected MCID threshold favoring high specificity at the expense of moderate sensitivity. An MCID of 11.5 points on the Mayo Wrist Score indicates a clinically meaningful and perceptible change, whereas more minor changes may not be recognized as significant improvements. The conceptual framework underlying MCID and patient-acceptable outcome thresholds has been widely discussed in the literature, emphasizing that clinically meaningful change extends beyond statistical significance and varies according to outcome instruments and patient populations [21-23].

For the QuickDASH, a change of approximately 14 points falls within the range previously reported for upper-extremity conditions (8-19 points) [24,25]. Similar findings were reported by Sorensen et al., who identified an MCID of 14 points for the QuickDASH [24]. Although the Mayo Wrist Score is less frequently used in research compared to the QuickDASH, it demonstrated a strong association with patient-perceived improvement in this study. Patients who reported being “much improved” following surgery met the SCB threshold of 13.5 points, with an excellent AUC of 0.91, indicating intense discrimination.

The overall improvement observed in both the QuickDASH and Mayo Wrist Scores is consistent with the literature on outcomes following carpal tunnel release. In the present study, 68 (93.2%) patients reported postoperative improvement, with the majority experiencing either substantial or moderate symptom relief. Mean QuickDASH scores improved by approximately 46.7 points, while Mayo Wrist Scores increased by approximately 42 points, supporting the effectiveness of surgical intervention in alleviating symptoms and improving function. These findings reinforce the importance of interpreting patient-reported outcomes based on clinically meaningful thresholds rather than relying solely on mean score changes.

MCID provides a standardized metric for evaluating and comparing treatment effectiveness across studies. For hand surgeons, MCID and SCB values facilitate interpretation of outcome measures, improve clinical decision-making, and help set realistic expectations for patients undergoing carpal tunnel release. Outcomes exceeding the MCID threshold suggest that surgery was successful from the patient’s perspective. However, patient perception of improvement may be influenced by several factors, including age, cultural background, baseline disease severity, demographic characteristics, comorbidities, occupation-related factors, and individual expectations.

Limitations

Several limitations of this study should be acknowledged. First, there was a loss to follow-up, as some patients who experienced marked improvement were discharged before completing the six-month evaluation. Although follow-up via phone was permitted, this may have influenced patient-reported outcomes. Second, CTS frequently affects both wrists; however, only the operated hand was assessed, with limited information available regarding the contralateral side. Third, the follow-up period was limited to six months, and functional outcomes may continue to evolve beyond this time frame [26,27]. Another consideration relates to the choice of outcome measures. In this study, the QuickDASH and Mayo Wrist Score were used because they are routinely employed in the participating institutions and allow comparison with broader upper-extremity outcome literature. While carpal tunnel-specific instruments such as the Boston Carpal Tunnel Questionnaire or Carpal Tunnel Syndrome-6 may provide more detailed symptom-specific assessment, they were not included in the present study protocol. Future studies may benefit from incorporating CTS-specific patient-reported outcome measures alongside general upper-extremity instruments to provide a more comprehensive evaluation of postoperative outcomes. Finally, variation in MCID calculation methods across studies limits direct comparison, highlighting the need for condition- and instrument-specific benchmarks.

Future studies should address these limitations by incorporating more extended follow-up periods, larger sample sizes, multicenter participation, and comparative analyses of different surgical techniques. Establishing standardized MCID and SCB values for commonly used hand outcome measures will further enhance the clinical applicability of patient-reported outcomes in hand surgery research. Although further studies with larger cohorts and longer follow-up are warranted, the results of this study support the use of Mayo Wrist Score MCID values and contextualized QuickDASH changes as practical tools for outcome assessment in hand surgery.

## Conclusions

In this study, the MCID for the Mayo Wrist Score following carpal tunnel release was identified as 11.5 points, while the SCB threshold was 13.5 points. These findings provide clinically interpretable benchmarks for evaluating patient-perceived improvement after surgery. Interpreting postoperative QuickDASH changes alongside these thresholds further supports the meaningful assessment of functional recovery. Together, these results help clarify the interpretation of patient-reported outcomes and may assist clinicians and researchers in evaluating treatment success following carpal tunnel release.
